# Effects of dietary fermented peony seed dreg on the laying performance, albumen quality, antioxidant capacity, and n-3 PUFA-enriching property of laying hens

**DOI:** 10.3389/fvets.2022.1109869

**Published:** 2023-01-12

**Authors:** Yi Wan, Ruiyu Ma, Renrong Qi, Jing Lu, Zaigui Wang, Qiugang Ma, Wei Liu, Junying Li, Yan Li, Kai Zhan

**Affiliations:** ^1^Anhui Key Laboratory of Livestock and Poultry Product Safety Engineering, Institute of Animal Husbandry and Veterinary Medicine, Anhui Academy of Agricultural Sciences, Hefei, China; ^2^College of Life Sciences, Anhui Agricultural University, Hefei, China; ^3^State Key Laboratory of Animal Nutrition, College of Animal Science and Technology, China Agricultural University, Beijing, China

**Keywords:** fermented peony seed dreg, laying performance, egg quality, serum parameters, n-3 polyunsaturated fatty acid

## Abstract

**Introduction:**

It is of great importance to seek agro-industrial byproducts that can serve as unconventional or alternative feedstuffs for poultry, especially those that are substitutive sources of n-3 polyunsaturated fatty acids (PUFAs), as this will contribute to alleviating feed shortage pressure and improving poultry performance and product quality. In this study, the effects of dietary fermented peony seed dreg (FPSD) on the production performance of hens and fatty acid composition of meat and egg yolk were evaluated.

**Methods:**

A total of 480 54-week-old Xinyang chickens were divided into 5 groups: control (basal diet); 5% peony seed dreg (PSD); and 5%, 7% and 9% FPSD, and each group consisted of 6 replicates with 16 birds per replicate. Production performances were recorded daily, and egg quality, serum parameters, antibody titers and fatty acid profile in the muscle and yolk were measured at 64 weeks of age.

**Results and discussion:**

Egg production, feed conversion ratio and egg albumen quality were improved (*p* < 0.05) by 5% and 7% FPSD groups compared to 5% PSD and the control diet. Immune status was not influenced by PSD, but antibody titres against H7N9 and ND were improved by FPSD diets during most of the experimental periods. Dietary 5% and 7% FPSD increased (*p* < 0.05) serum high density lipoprotein concentrations and glutathione peroxidase actives. Total n-3 polyunsaturated fatty acids (PUFA) in meat and yolk increased gradually, while the total saturated fatty acids (SFA) and the n-6/n-3 PUFA ratio decreased gradually with increasing dietary FPSD levels. In conclusion, up to 7% dietary FPSD has the potential use to be utilized as a supplement in Xinyang laying hen diets to produce n-3 PUFA-enriched meat and eggs and positively affect production performance and health status.

## 1. Introduction

Omega-3 polyunsaturated fatty acids (n-3 PUFAs) play important roles in anti-inflammation, neurogenesis, pregnancy, and neonatal health (1, 2). The three major n−3 PUFAs are α-linolenic acid (C18:3n−3, ALA), eicosapentaenoic acid (C20:5n−3, EPA), and docosahexaenoic acid (C22:6n−3, DHA). Health benefits are mainly ascribed to EPA and DHA rather than ALA, as the conversion of ALA to EPA and further to DHA is very limited and inefficient in the human body (3). High levels of dietary DHA and EPA have been associated with lower rates of coronary heart disease; arrhythmias; atherosclerosis; diabetes; and cancers of the breast, prostate, and colon (4). Therefore, food enrichment is probably the best long-term solution to boost the intake of these unsaturated fatty acids.

Eggs are an integral part of the human diet not only as a food but also as an ingredient in many dishes. Recent studies have shown that n-3 PUFA enrichment of egg yolk through dietary supplementation of laying hens with ingredients such as rubber seed oil and sugar beet pulp is effective (5, 6). In the past few years, fish oil and flaxseed have been the main sources of n-3 PUFA in poultry feed. Nevertheless, flaxseed was reported to be effective in enriching eggs with ALA but not EPA or DHA due to the limited ability of birds to metabolically convert ALA into EPA and DHA (7). Fishy and rancid flavors of eggs could be caused by dietary fish oil, which limits the wide acceptance of this ingredient in layer diets (8). Furthermore, the increasing demand for poultry meat production makes breeders strive to increase the growth rate of birds, and the rapid growth of poultry has adverse effects on meat quality (9), especially in terms of increased abdominal fat and reduced PUFAs (10); this in turn affects its nutrition and flavor, which are closely related to human health. In addition, the shortage of feed and rising price of conventional feed ingredients are becoming concerns, indicating a serious challenge for poultry production in the next few years. The continuous decline in the laying rate, egg quality and immune function of chickens during the later laying period has badly hindered the profits of poultry farmers (11), forcing layer farmers to continually seek ways to boost their profits by increasing production performance, enhancing egg and meat quality, and maintaining flock health, especially during the later laying period. Hence, it is of great importance to seek agro-industrial byproducts that can serve as unconventional or alternative feedstuffs for poultry, especially those that are substitutive sources of n-3 PUFAs, as this will contribute to alleviating feed shortage pressure and improving poultry performance and product quality during the late laying period.

The tree peony *Paeonia ostiii* “Feng Dan? is well-known for its multifaceted properties and diverse uses; belongs to the Jiangnan cultivars; is mainly distributed in the city of Tongling, China; and has a long history of artificial cultivation in many other countries (12, 13). Seeds of peony are rich in oil, and peony seed oil (PSO) is one of the functional foods that has been widely consumed in China; PSO was reported to contain abundant polyunsaturated fatty acids (PUFAs, >90%), especially α-linolenic acid (ALA, >40%), and play an important role in oxidation resistance and fighting against various diseases (14–16). Peony seed dreg (PSD) is a byproduct of the process of extracting oil from peony seeds, with a yield of 25 ~ 30% and a protein content of approximately 20%; PSD is rich in PUFAs, polysaccharides and a variety of essential amino acids (14, 17). Some peptides and polysaccharides in PSD were reported to exhibit appreciable antioxidative and anticancer properties from a functional perspective (17, 18), and stilbenoid compounds (trans-resveratrol) extracted from PSD displayed health benefits in humans (19, 20). These studies indicated important potential industrial applications of PSD in functional feed. Unfortunately, due to the lack of technical standards, large amounts of PSD are discarded every year, resulting in resource waste and environmental pollution. This situation may be due to the poor palatability of untreated dreg material; most proteins displayed low solubility and dispersive properties in water when compared to those of animal proteins, despite containing many useful compounds. However, microbial fermentation could be an effective way to reduce natural antinutritional factors and improve the palatability and utilization of PSD (21). In light of this possibility, fermented peony seed dreg (FPSD) could be considered a potential feedstuff for poultry production.

To our knowledge, studies regarding the influence of PSD and FPSD on the production performance and physicochemical properties of muscle and eggs in laying hens remain scarce. It is hypothesized that PSD and FPSD will impact the production performance, antioxidant capacity and immune performance of laying hens and will greatly influence its meat and egg quality. Therefore, the purpose of this experiment was to study the effects of dietary PSD and FPSD on the laying performance, egg quality, serum biochemical indices and the fatty acid composition of muscle and eggs in Xinyang laying hens during the late laying period.

## 2. Materials and methods

All animal procedures of this experiment were approved by the Committee for the Care and Use of Experimental Animals at Anhui Academy of Agricultural Science under permit No. A11-CS06.

### 2.1. Preparation of FPSD

PSD was obtained from Tongling Fengdan Planting Technology Development Co., Ltd., Tongling, China. The *Lactobacillus acidophilus* (ACCC11073), *Bacillus subtilis* (CICC10275), and *Saccharomycetes* (CICC1005) used in this study were obtained from the China Center of Industrial Culture Collection (CICC). The method of PSD fermentation was described previously (22). Dried PSD was soaked with distilled water for 60 min and then cooked in a steam tank at 65°C for 1 h. Cooked PSD was cooled to room temperature (18–20°C) for 1 h, inoculated with *Bacillus subtilis* (6 × 107 cfu/g), *Lactobacillus acidophilus* (4.25 × 107 cfu/g), and *Saccharomycetes* (5.4 × 105 cfu/g); mixed; and fermented in a bed-packed incubator for 48 h. After fermentation, fresh fermented samples were dried at 50 to 60°C for 3 d. The dried samples were ground and refrigerated until they were mixed in the diets.

### 2.2. Laying hens and dietary treatments

The Xinyang chicken originated in South China, is used as dual-purpose breed and is one of the most popular local laying hen breeds in China, which is characterized by its black plumage and bone. A total of 480 54-week-old healthy Xinyang laying hens with similar body weight (1,820.47 ± 65.22 g) were obtained from Shanghai Poultry Breeding Co., Ltd., Shanghai, China, and were randomly allotted to five experimental diets, each including 6 replicates with 16 birds per replicate. The birds were kept in a three-tier battery cage, and each cage (width 430, length 500, height 450 mm) included 2 birds. Eight sequential cages with one diet trough were arranged as a replicate, and all replicates were equally distributed in different spatial directions. A preliminary study was conducted for 2 weeks, and the formal experiment was performed from weeks 56 to 64. Water and feed were supplied for *ad libitum* consumption. The average indoor air temperature and relative humidity were controlled at 21.5°C and 60%, respectively, during the trial period.

The dietary composition and nutrient levels were formulated to meet the recommended nutrient requirements stipulated by the “NY/T33-2004 feeding standard of chicken”. PSD or FPSD was added to corn–soybean meal diets at the expense of corn and soybean to produce experimental diets containing 0 (control), 5 PSD, 5 FPSD, 7 FPSD, and 9% FPSD. PSD and FPSD were provided by Tongling Planting Technology Co., Ltd. (Anhui, China). The experimental diets had similar nutrient levels, which are shown in Table 1. The nutrient contents and fatty acid compositions of PSD and FPSD were measured and are shown in Table 2.

**Table 1:** Ingredients and nutrient levels of experimental diets for laying hens (as-fed basis).

**Items**	**Control**	**5% PSD**	**FPSD supplemental levels (%)**		
			**5**	**7**	**9**
**Ingredients (%)**					
Corn	62.02	60.8	58.61	57.55	56.24
Soybean meal	24.48	20.7	22.21	20.99	19.87
Soybean oil	0	0	0.68	0.96	1.39
PSD	0	5	0	0	0
FPSD	0	0	5	7	9
Limestone	8.5	8.5	8.5	8.5	8.5
Premix^a^	5	5	5	5	5
Total	100	100	100	100	100
**Analyzed nutrient content**					
ME (MJ/kg)^b^	10.84	10.84	10.84	10.84	10.84
Crude protein (%)	15.56	15.49	15.52	15.53	15.54
Calcium (%)	4.43	4.45	4.41	4.42	4.41
Total phosphorus (%)	0.44	0.43	0.42	0.41	0.43
Threonine (%)	0.59	0.55	0.56	0.60	0.58
Methionine (%)	0.27	0.27	0.28	0.28	0.27
Methionine + Cysteine (%)	0.67	0.68	0.69	0.68	0.68
Lysine (%)	0.82	0.71	0.72	0.81	0.78

a Provided the following per kilogram of feed: vitamin A, 10,000IU; vitamin B1, 1.8 mg; vitamin B2, 5.0 mg; vitamin B5, 12 mg; vitamin B6, 3.75 mg; vitamin B12, 0.025 mg; vitamin D3, 3000IU; vitamin E, 20IU; vitamin K3, 2.0 mg; biotin, 0.04 mg; folic acid, 0.6 mg; Cu, 8 mg; Fe, 80 mg; Mn, 100 mg; Zn, 75 mg; I, 0.15 mg; Se, 0.10 mg; and Choline chloride, 500 mg.

b Values were calculated according to the MEn of feedstuffs for poultry provided by the “NY/T33-2004 feeding standard of chicken.”

**Table 2:** The nutrient contents and fatty acids compositions of PSD and FPSD.

**Nutrient content (%)**	**PSD**	**FPSD**	**Amino acids^a^ (%)**	**PSD**	**FPSD**	**Fatty acids (mg/g)**	**PSD**	**FPSD**
pH	6.6	4.9	Indispensable			C12:0	0.14	0.04
Moisture	7.5	32.3	Lysine	0.83	0.98	C14:0	0.05	0.04
crude protein	23.18	26.25	Tryptophan	0.54	0.55	C16:0	5.17	4.87
crude fiber	8.7	5.6	Threonine	0.74	0.95	C18:0	1.49	1.21
crude fat	5.6	4.8	Phenylalanine	0.80	0.84	C20:0	0.14	0.15
Calcium	0.24	0.30	Valine	1.23	1.61	C21:0	0.04	0.03
Phosphorus	0.51	0.61	Isoleucine	0.94	0.91	C22:0	0.05	0.02
Ash	1.19	1.31	Leucine	1.64	1.88	Total SFA	7.08	6.36
			Histidine	0.52	0.50	C16:1	0.07	0.06
			Dispensable			C18:1n-9	12.91	16.16
			Serine	0.95	0.96	C20:1	0.16	0.16
			Glutamate	4.85	5.17	C22:1	0.02	0.03
			Proline	0.86	0.92	Total MUFA	13.16	16.41
			Glycine	1.14	1.11	C18:2n-6 (LA)	12.62	17.88
			Alanine	1.10	1.13	C20:3n-6 (DGLA)	0.05	0.09
			Cysteine	0.43	0.66	C20:4n-6 (AA)	0.04	0.09
			Aspartate	2.10	2.64	n-6 PUFA^b^	12.71	18.06
			Methionine	0.40	0.45	C18:3n-3 (ALA)	18.67	26.43
			Arginine	1.39	1.65	C20:5n-3 (EPA)	0.05	0.11
						C22:6n-3 (DHA)	0.03	0.10
						n-3 PUFA^c^	18.75	26.64
						Total PUFA	31.46	44.70

PSD, peony seed dreg; FPSD, fermented peony seed dreg; SFA, saturated fatty acid; MUFA, monounsaturated fatty acid; PUFA, polyunsaturated fatty acid; LA, linoleic acid; DGLA, dihomo γ-linolenic acid; ALA, α-linolenic acid; AA, arachidonic acid; EPA, eicosapentaenoic acid; DHA, docosahexaenoic acid.

a On a % dry matter basis.

b n-6 PUFA = C18:2n-6 + C20:3n-6 + C20:4n-6.

c n-3 PUFA = C18:3n-3 + C20:5n-3 + C22:6n-3.

### 2.3. Production performance

Eggs were collected, counted, and weighed on a cage basis every day to calculate the laying rate, average egg weight and the ratio of abnormal eggs. Egg mass was calculated by multiplying the hen-day laying rate by the average egg weight. The average daily feed intake (ADFI) and the feed conversion ratio (FCR) were recorded/calculated to analyze production performance. The FCR was calculated as the ratio of feed intake per unit of egg mass.

### 2.4. Measurement of egg quality

At 64 weeks of age, 48 eggs from each group (8 per replicate) were randomly collected for the determination of egg quality. All eggs were kept in the same storage room, and egg quality measurements were completed on the day of collection. Measurements of egg length and width were taken with a digital caliper to the nearest 0.01 mm, and the egg shape index was the ratio of length to width. Egg weight was measured using an electronic scale with an accuracy of 0.01 g. Shell strength was measured with an eggshell force gauge (EGG-0503, Robotmation Co., Ltd., Tokyo, Japan). Shell thickness was measured at the eggshell equator in three places using a micrometer gauge (FHK Co., Ltd., Tokyo, Japan). Yolk color, albumen height and Haugh unit (HU) were measured using an automatic egg multitester (EMT-5200, Robotmation Co., Ltd., Tokyo, Japan). Yolks were separated from the albumen, and then the chalazae were carefully removed from the yolk before weighing the yolk. The yolk percentage was calculated based on the following equation: yolk percentage = 100 × (yolk weight/egg weight).

### 2.5. Measurements of serum biochemical parameters and antibody titers

At 64 weeks of age, 48 birds from each group (8 per replicate) were selected for blood sampling. A 4-mL blood sample was collected from the wing vein of the chickens into 2 heparinized tubes (2 mL in each tube). The time between securing the bird and obtaining the blood sample did not exceed 120 s. Samples were placed in an ice bath immediately after collection and then transported to the laboratory for processing. Blood serum was separated by centrifugation for 10 min (3000 × g) at 4°C and stored at−20°C until analysis. The total cholesterol (T-CH) level, triglyceride (TG) level, high-density lipoprotein (HDL), and low-density lipoprotein (LDL) levels, malondialdehyde (MDA) level, serum glutathione peroxidase (GSH-Px) activity, and superoxide dismutase (SOD) activity were determined by commercial analytical kits (Sigma, Thermo Fisher Scientific, Shanghai, China) with an autoanalyzer (Hitachi Ltd., Tokyo, Japan). Antibody titers against the avian influenza viruses H5N1, H7N9, and H9N2 and against Newcastle disease (ND) virus were determined with enzyme-linked immunosorbent assay (ELISA) kits (Mlbio Biotech Co., Shanghai, China) according to the manufacturer′s protocol. Antibody titer data were logarithmically transformed (base 2) prior to analysis.

### 2.6. Measurements of fatty acid profile in the muscle and yolk

At 64 weeks of age, 18 birds from each group (3 per replicate) were selected and sacrificed by CO_2_ suffocation. The breast and thigh muscle samples were collected, cut into small samples and then frozen within 10 min postmortem. Three eggs from each replicate were collected, and yolks were separated using an egg separator and then frozen at−20°C for further determination. For fatty acid analyses, muscle and yolk samples were freeze-dried by a Freeze dryer (LG-06, Songyuan Co., Ltd., Beijing, China) and smashed by a pulverizer (FS-200, Tianhe Machinery Co.Ltd., Shanghai, China), prior to being passed through a 40-mesh sieve. The fatty acid composition of the samples was measured according to the method described in a previous study (23). The fatty acid composition of the samples was determined using gas chromatography (Agilent 6,890 Series Systems, with FID detector, Agilent Technologies Inc., Beijing, China). An automatic sampler with a flow rate of 2.0 mL/min was used to inject the sample (1 μL) onto the DB-23 column (60 μm × 250 μm × 0.25 μm), and the temperature was increased to 260°C. The carrier gas was helium at a flow rate of 2 mL/min. The calibration and peak determinations were based on authentic standard fatty acids from Sigma–Aldrich (St Louis, United States). The mean level of each fatty acid was used to calculate the total saturated fatty acid (SFA), total monounsaturated fatty acid (MUFA), and total PUFA. The results are shown as milligrams per gram of sample powder.

### 2.7. Statistical analysis

The results were analyzed by a one-way analysis of variance (ANOVA) using SAS (SAS Institute Inc., Cary, NC) software. Data are shown as the means and SEM. Tukey′s multiple comparison was used to test the significance of the differences between treatment means; significance was declared at *p* < 0.05.

## 3. Results

### 3.1. Production performance and egg quality traits

The production performance and egg quality traits of the laying hens fed different dietary supplements are presented in Table 3. During the whole experimental period (56–64 weeks of age), the laying rate, FCR and abnormal egg rate of birds fed the FPSD diets were superior to those fed the 5% PSD and control diets, of which birds fed the 5 and 7% FPSD diets had higher laying rate (74.53 and 75.82%) and FCR (2.63 and 2.61) and lower abnormal egg rate (0.14 and 0.09%). There were no significant differences in egg weight among the five treatment groups, while 5 and 7% FPSD supplementation slightly increased the egg weight (*p* > 0.05). Compared to the control, yolk color was significantly enhanced in the 5% PSD and FPSD groups (*p* < 0.05), whereas the yolk proportion was slightly decreased in the 5% PSD and FPSD groups (*p* >0.05). Additionally, the 5 and 7% FPSD supplementation increased the albumen height and improved the Haugh unit, especially for the 7% FPSD group, which improved by 12.74% (4.78 mm *vs*. 4.24 mm) and 6.83% (66.62 *vs*. 62.36), respectively, compared with the control group. The Haugh unit was also slightly increased in the 5% PSD group compared with that in the control. There were no significant effects observed for other traits among the five treatment groups (*p* > 0.05).

**Table 3:** Effect of dietary PSD and FPSD on the overall performance of laying hens (56–64 weeks of age)^1^.

**Items**	**Control**	**5% PSD**	**FPSD supplemental levels (%)**			**SEM**	***P*-value**
			**5**	**7**	**9**		
**Production performance**							
Laying rate (%)	72.22^a^	72.33^a^	74.53^b^	75.82^b^	72.69^a^	1.561	0.001
Egg mass (g/bird/d)	37.52	37.55	39.165	40.43	37.68	1.343	0.374
ADFI (g/bird/d)	102.65	101.30	103.20	101.06	101.46	1.814	0.793
FCR	2.75^b^	2.73^b^	2.63^a^	2.61^a^	2.71^b^	0.121	0.016
Abnormal egg (%)	0.35^b^	0.30^b^	0.13^a^	0.09^a^	0.14^a^	0.023	< 0.001
**Egg quality trait**							
Egg weight (g)	51.96	51.98	52.59	53.37	51.83	0.867	0.517
Egg shape index	1.34	1.33	1.37	1.36	1.34	0.061	0.878
Shell strength (kg/cm^2^)	3.87	3.85	4.01	4.03	3.75	0.128	0.392
Shell thickness (mm)	0.32	0.34	0.31	0.34	0.33	0.036	0.653
Yolk color	5.00^a^	5.30^b^	5.37^b^	5.58^c^	5.62^c^	0.159	0.002
Yolk proportion (%)	31.76	30.69	31.16	30.62	30.80	1.220	0.126
Albumen height (mm)	4.24^a^	4.32^a^	4.53^ab^	4.78^b^	4.18^a^	0.147	0.014
Haugh unit	62.36^a^	63.30^a^	64.32^ab^	66.62^b^	62.27^a^	1.138	0.007

PSD, peony seed dreg; FPSD, fermented peony seed dreg; ADFI, average daily feed intake; FCR, feed conversion ratio.

a, b, c within a row, values with different superscripts indicate a significant difference (p < 0.05).

1 Each value represents the mean of 6 replicates (n = 6).

### 3.2. Immune response

**Figure 1:**
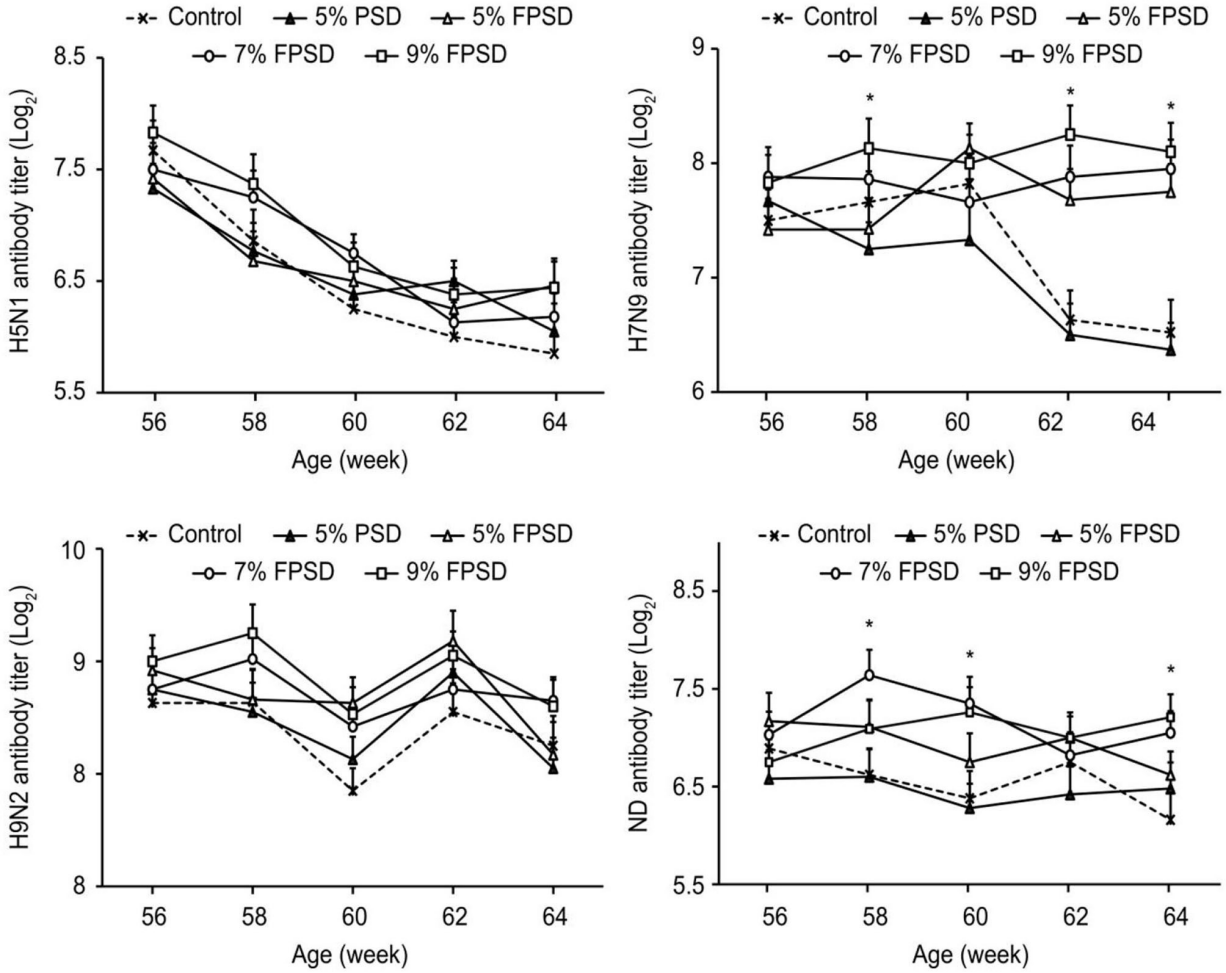
Effect of PSD and FPSD on the antibody titer of H5N1, H7N9, H9N2, and ND of laying hens (56 to 64 weeks of age)^1^. PSD, peony seed dreg; FPSD, fermented peony seed dreg; *Means with asterisk superscripts within each period are significantly different (*p* < 0.05). ^1^Each value represents the mean of 6 replicates (*n* = 6).

The immune performance of the laying hens fed different dietary supplements is presented in Figure 1. The antibody titer against H7N9 was significantly increased in the birds from the FPSD groups compared with those from the 5% PSD and control groups (*p* < 0.05) at 62–64 weeks of age. The antibody titer against ND was increased in the birds from the 7 and 9% FPSD groups compared with those from the 5% PSD and control groups (*p* < 0.05) from 58 to 64 weeks of age (except at 62 weeks). However, compared with the control, birds fed diets supplemented with 5% PSD exhibited the lowest levels of H7N9 (*p* < 0.05) from 58 to 64 weeks of age and lower levels of ND (*p* < 0.05) from 58 to 62 weeks of age.

### 3.3. Serum biochemical index

The serum biochemical parameters of the laying hens fed different dietary supplements are presented in Table 4. The serum concentrations of TGs were slightly lower in the 5% PSD and FPSD groups than in the control group and were further decreased in the 7 and 9% FPSD groups. The serum concentration of HDL was significantly increased in the 5 and 7% FPSD groups (*p* < 0.05) but was significantly decreased in the 5% PSD group (*p* < 0.05) compared to that in the control group. The activities of antioxidant enzymes, namely, GSH-Px and SOD, were higher in the 5 PSD and 5, 7, and 9% FPSD groups than in the control group, and the GSH-Px enzyme activity was significantly enhanced (*p* < 0.05), improving by 8.50, 18.83, 22.47, and 9.83%, respectively. In addition, the level of MDA was significantly decreased in the 5 and 7% FPSD groups compared to that in the control group (*p* < 0.05).

**Table 4:** Effect of dietary PSD and FPSD on serum biochemical parameters in laying hens after 8 weeks of feeding.^1^

**Items**	**Control**	**5% PSD**	**FPSD supplemental levels (%)**			**SEM**	***P*-value**
			**5**	**7**	**9**		
T-CH (μmol/L)	972.87	971.03	1,003.33	981.18	982.72	7.822	0.394
TG (μmol/L)	360.55	353.41	348.82	335.34	347.49	4.659	0.772
HDL (μmol/L)	115.80^b^	109.97^a^	124.00^c^	122.11^c^	117.47^b^	2.114	0.011
LDL (μmol/L)	230.82	233.91	225.14	228.12	225.21	3.343	0.357
GSH-Px (ng/L)	565.04^a^	613.08^b^	671.45^c^	692.02^c^	620.59^b^	5.657	< 0.001
SOD (pg/mL)	37.18	36.96	38.79	38.72	37.68	1.503	0.513
MDA (nmol/L)	15.18^c^	14.79^bc^	14.08^a^	14.47^b^	15.00^bc^	0.721	0.026

T-CH, the total cholesterol; TG, triglyceride; HDL, high-density lipoprotein; LDL, low-density lipoprotein; GSH-Px, glutathione peroxidase; SOD, superoxide dismutase; MDA, malondialdehyde.

a, b, c within a row, values with different superscripts indicate a significant difference (p < 0.05).

1 Each value represents the mean of 6 replicates (n = 6).

### 3.4. Muscle fatty acid content

The fatty acid contents of breast and thigh muscle for laying hens fed different dietary supplements are presented in Table 5. Regarding SFA, the contents of tetradecanoic acid (C14:0) and palmitic acid (C16:0) in breast muscle and the content of C16:0 in thigh muscle were significantly lower in all experimental treatment groups than in the control group (*p* < 0.05), which contributed to the significant decrease in the content of total SFA in muscle for all FPSD-supplemented treatments (*p* < 0.05). Regarding MUFAs, the content of methyl oleate (C18:1n-9) in the breast muscle was increased in the 5% FPSD group (*p* < 0.05) but was decreased in the 7 and 9% FPSD groups (*P* < 0.05), as well as significantly decreased in the thigh muscle in the 9% FPSD group (*p* < 0.05). Supplementation with 5% PSD or any level of FPSD decreased the content of linoleic acid (C16:1) and erucic acid (C22:1) in thigh muscle compared to the control condition, and the content of total MUFAs was the lowest in the 9% FPSD group (*p* < 0.05). Regarding PUFAs, all FPSD-supplemented diets resulted in a significantly increased content of linoleic acid (C18:2n-6) in both the breast and thigh muscle (*p* < 0.05), and supplementation with 5% PSD as well as 7 and 9% FPSD increased the content of dihomo γ-linolenic acid (DGLA, C20:3n-6) and arachidonic acid (AA, C20:4n-6) in breast muscle (*p* < 0.05). A similar trend was observed for the content of α-linolenic acid (ALA, C18:3n-3) in the muscle as well as the content of docosahexaenoic acid (DHA, C22:6n-3) in breast muscle, which were higher in all experimental groups (*p* < 0.05) than in the control group. Finally, 5% PSD and FPSD diets increased the content of n-6 PUFA and n-3 PUFA (*p* < 0.05) and decreased the n-6/n-3 PUFA ratio (*p* < 0.05) in the muscle compared to the control, where the content of n-6 PUFA, n-3 PUFA and total PUFA increased linearly with increasing dietary FPSD levels.

**Table 5:** Effect of dietary PSD and FPSD on fatty acid content in breast and thigh muscle of laying hens after 8 weeks of feeding^1^ (mg/g).

**Items**	**Control**	**5% PSD**	**FPSD supplemental levels (%)**			**SEM**	***P*-value**
			**5**	**7**	**9**		
**Breast muscle**							
C12:0	0.07	0.05	0.04	0.07	0.06	0.001	0.622
C14:0	0.61^b^	0.48^a^	0.51^a^	0.51^a^	0.51^a^	0.015	0.013
C16:0	23.40^b^	21.26^a^	21.11^a^	20.79^a^	20.82^a^	0.176	0.006
C18:0	7.35	8.49	6.92	6.68	6.74	0.112	0.104
C20:0	0.13	0.14	0.14	0.15	0.19	0.011	0.110
C21:0	0.21	0.27	0.19	0.26	0.29	0.027	0.349
C22:0	0.07	0.12	0.07	0.11	0.15	0.011	0.287
Total SFA	31.86^b^	30.82^ab^	28.99^a^	28.58^a^	28.76^a^	0.224	< 0.001
C16:1	3.60	3.83	3.32	3.71	3.10	0.082	0.251
C18:1n-9	36.65^b^	34.37^b^	38.11^c^	32.79^a^	31.97^a^	0.326	< 0.001
C20:1	0.27	0.29	0.26	0.24	0.24	0.025	0.928
C22:1	0.05	0.06	0.04	0.07	0.08	0.001	0.562
Total MUFA	40.58^b^	38.50^a^	41.72^b^	36.82^a^	35.41^a^	0.419	< 0.001
C18:2n-6 (LA)	23.77^a^	24.54^a^	26.97^b^	26.68^b^	28.19^c^	0.317	< 0.001
C20:3n-6 (DGLA)	0.24^a^	0.37^b^	0.19^a^	0.38^b^	0.37^b^	0.038	0.009
C20:4n-6 (AA)	3.04^a^	5.38^b^	2.79^a^	4.31^b^	4.80^b^	0.096	< 0.001
n-6 PUFA^2^	27.07^a^	30.31^b^	29.96^ab^	31.39^bc^	33.37^c^	0.373	< 0.001
C18:3n-3 (ALA)	0.34^a^	0.67^b^	0.66^b^	0.69^b^	0.93^c^	0.073	< 0.001
C20:5n-3 (EPA)	0.01	0.01	0.01	0.03	0.03	0.001	0.513
C22:6n-3 (DHA)	0.82^a^	1.41^b^	1.11^b^	1.13^b^	1.33^b^	0.029	0.003
n-3 PUFA^3^	1.17^a^	2.10^cd^	1.78^b^	1.85^bc^	2.31^d^	0.075	< 0.001
n-6/n-3	22.89^c^	14.61^a^	16.78^b^	16.86^b^	14.51^a^	0.180	< 0.001
Total PUFA	28.25^a^	32.43^b^	31.75^b^	33.25^b^	35.69^c^	0.387	< 0.001
**Thigh muscle**							
C12:0	0.05	0.04	0.04	0.11	0.05	0.001	0.201
C14:0	0.69^b^	0.59^b^	0.61^b^	0.66^b^	0.49^a^	0.056	0.005
C16:0	23.31^c^	19.98^b^	20.40^b^	20.51^b^	18.75^a^	0.267	< 0.001
C18:0	6.46	6.85	6.86	6.93	6.01	0.135	0.094
C20:0	0.15	0.16	0.15	0.17	0.21	0.014	0.407
C21:0	0.19	0.20	0.20	0.23	0.18	0.016	0.356
C22:0	0.15	0.09	0.07	0.12	0.09	0.011	0.278
Total SFA	31.01^d^	27.91^bc^	28.33^bc^	28.73^c^	25.78^a^	0.289	< 0.001
C16:1	3.19^c^	1.84^ab^	1.95^ab^	2.76^bc^	1.57^a^	0.136	0.003
C18:1n-9	38.52^cd^	36.99^ab^	37.46^bc^	37.11^bc^	35.39^a^	0.322	0.005
C20:1	0.30	0.34	0.32	0.33^b^	0.34	0.021	0.369
C22:1	0.08^c^	0.04^ab^	0.04^ab^	0.07^bc^	0.03^a^	0.001	0.011
Total MUFA	42.10^b^	39.21^b^	39.77^b^	40.27^b^	37.33^a^	0.407	0.003
C18:2n-6 (LA)	25.45^a^	28.96^bc^	28.16^b^	28.12^b^	31.71^c^	0.248	< 0.001
C20:3n-6 (DGLA)	0.19	0.16	0.16	0.19	0.13	0.012	0.259
C20:4n-6 (AA)	1.64	1.80	1.85	1.94	1.97	0.045	0.602
n-6 PUFA	27.28^a^	30.92^bc^	30.17^b^	30.25^b^	33.81^c^	0.289	< 0.001
C18:3n-3 (ALA)	0.83^a^	1.08^b^	1.09^b^	1.25^c^	1.39^cd^	0.038	< 0.001
C20:5n-3 (EPA)	0.04	0.02	0.01	0.02	0.02	0.001	0.872
C22:6n-3 (DHA)	0.16	0.27	0.28	0.29	0.24	0.001	0.391
n-3 PUFA	1.04^a^	1.37^b^	1.40^b^	1.57^bc^	1.66^c^	0.013	0.001
n-6/n-3	26.51^b^	22.39^a^	21.59^a^	19.41^a^	20.36^a^	0.225	0.011
Total PUFA	28.32^a^	32.31^bc^	31.55^b^	31.82^b^	35.47^c^	0.336	< 0.001

PSD, peony seed dreg; FPSD, fermented peony seed dreg; SFA, saturated fatty acid; MUFA, monounsaturated fatty acid; PUFA, polyunsaturated fatty acid; LA, linoleic acid; DGLA, dihomo γ-linolenic acid; ALA, α-linolenic acid; AA, arachidonic acid; EPA, eicosapentaenoic acid; DHA, docosahexaenoic acid.

a, b, c, d within a row, values with different superscripts indicate a significant difference (p < 0.05).

1 Each value represents the mean of 6 replicates (n = 6).

2 n-6 PUFA = C18:2n-6 + C20:3n-6 + C20:4n-6.

3 n-3 PUFA = C18:3n-3 + C20:5n-3 + C22:6n-3.

### 3.5. Yolk fatty acid content

The yolk fatty acid contents of the laying hens fed different dietary supplements are presented in Table 6. Compared with the control group, the total SFA content in yolk was lower in all experimental treatment groups (*p* < 0.05), which was due to the decreased content of C14:0 and C16:0. In addition, the contents of C16:1 and C18:1n-9 were decreased in all FPSD groups compared to in the control group, where the total MUFA content was significantly lower (*p* < 0.05). In terms of PUFAs, the contents of LA, ALA, and DHA in yolk increased linearly (*p* < 0.05) as the dietary level of FPSD increased gradually, which contributed to the gradual increase in the contents of n-6 PUFA and n-3 PUFA, improving by 43.05% (12.73 mg vs. 18.21 mg) and 117.76% (1.07 mg vs. 2.33 mg) in the 9% FPSD group, respectively. In addition, the contents of ALA, DHA, and total n-3 PUFA in yolk were increased by 5% PSD supplementation. Finally, dietary FPSD reduced the total SFA and MUFA contents and improved the total PUFA contents in yolk, particularly with regard to n-3 and n-6 PUFAs as well as their ratio.

**Table 6:** Effect of dietary PSD and FPSD on fatty acid content in egg yolks of laying hens after 8 weeks of feeding^1^ (mg/g).

**Items**	**Control**	**5% PSD**	**FPSD supplemental levels (%)**			**SEM**	***P* value**
			**5**	**7**	**9**		
C12:0	0.01	0.01	0.01	0.01	0.01	0.000	0.978
C14:0	0.43^b^	0.34^a^	0.33^a^	0.35^a^	0.31^a^	0.028	0.037
C16:0	27.57^b^	25.81^a^	25.54^a^	25.56^a^	24.91^a^	0.357	0.022
C18:0	8.30	7.86	8.52	8.23	8.14	0.179	0.325
C20:0	0.09	0.13	0.08	0.07	0.10	0.002	0.470
C21:0	0.20	0.21	0.20	0.21	0.22	0.022	0.659
C22:0	0.05	0.03	0.03	0.03	0.03	0.001	0.580
Total SFA	36.23^b^	34.40^a^	34.73^a^	34.46^a^	33.72^a^	0.445	0.038
C16:1	4.11^a^	3.61^ab^	3.21^ab^	3.20^ab^	2.76^b^	0.082	< 0.001
C18:1n-9	44.79^ab^	46.98^a^	43.16^ab^	42.18^b^	42.35^b^	0.518	0.007
C20:1	0.25^a^	0.32^b^	0.25^a^	0.25^a^	0.26^a^	0.016	0.029
C22:1	0.06	0.05	0.04	0.04	0.05	0.001	0.873
Total MUFA	49.22^b^	50.97^b^	46.66^a^	45.68^a^	45.41^a^	0.670	0.005
C18:2n-6 (LA)	10.32^a^	10.41^a^	14.33^b^	15.25^b^	16.03^b^	0.356	< 0.001
C20:3n-6 (DGLA)	0.16	0.16	0.16	0.17	0.17	0.015	0.661
C20:4n-6 (AA)	2.26	1.97	2.28	2.12	2.02	0.031	0.456
n-6 PUFA^3^	12.73^a^	12.53^a^	16.74^b^	17.50^bc^	18.21^bc^	0.277	< 0.001
C18:3n-3 (ALA)	0.27^a^	0.53^b^	0.63^bc^	0.77^c^	0.93^d^	0.067	< 0.001
C20:5n-3 (EPA)	0.02	0.02	0.01	0.02	0.02	0.001	0.314
C22:6n-3 (DHA)	0.77^a^	1.24^b^	1.20^b^	1.23^b^	1.37^b^	0.071	< 0.001
n-3 PUFA^4^	1.07^a^	1.80^b^	1.85^b^	2.02^bc^	2.33^cd^	0.086	< 0.001
n-6/n-3	12.03^b^	7.12^a^	9.13^a^	8.59^a^	7.76^a^	0.349	< 0.001
Total PUFA	13.79^a^	14.32^a^	18.58^b^	19.52^b^	20.52^b^	0.421	< 0.001

PSD, peony seed dreg; FPSD, fermented peony seed dreg; SFA, saturated fatty acid; MUFA, monounsaturated fatty acid; PUFA, polyunsaturated fatty acid; LA, linoleic acid; DGLA, dihomo γ-linolenic acid; ALA, α-linolenic acid; AA, arachidonic acid; EPA, eicosapentaenoic acid; DHA, docosahexaenoic acid.

^a, b, c, d^ within a row, values with different superscripts indicate a significant difference (p < 0.05).

a Each value represents the mean of 6 replicates (n = 6).

b n-6 PUFA = C18:2n-6 + C20:3n-6 + C20:4n-6.

c n-3 PUFA = C18:3n-3 + C20:5n-3 + C22:6n-3.

## 4. Discussion

There are various useful bioactive constituents in peony seed dreg, among which proteins, PUFAs and polysaccharides are the main active components (20). Therefore, the application effect and value of the addition of PSD and FPSD to animal feed is expected and has potential benefits. The current results revealed that dietary PSD and FPSD could affect the production traits of laying hens, although not all indicators were significantly influenced. Compared to the control group, the laying rate, FCR and normal egg rate in the FPSD groups were improved, especially for the 5 and 7% FPSD supplementation, while 5% PSD slightly improved the laying rate and FCR, which indicated that appropriate dietary FPSD provided more beneficial effects than PSD and could enhance the conversion of digested feed into eggs. In addition, the albumen height and Haugh unit of eggs were also increased in the 5 and 7% FPSD groups compared to in the control and 5% PSD groups, which showed a higher albumen quality. The health-promoting effects of FPSD may be associated with its fermented microbe and bioactive constituents, which could promote digestion and absorption and be conducive to growth. The microorganisms used for PSD fermentation are probiotics, such as *Lactobacillus acidophilus, Bacillus subtilis*, and *Saccharomycetes*, which can positively affect production performance (24). These results were similar to the study reported by Ashayerizadeh et al. (25), who found that broiler chicken diets containing fermented rapeseed meal (fermented by *Lactobacillus, Bacillus subtilis*, and *Aspergillus niger*) resulted in better weight gain, feed intake, and FCR than those containing rapeseed meal. Fermentation of PSD with probiotics could promote protein and starch digestibility and improve the palatability of feed (26), which could contribute to protein secretion in the magnum of the oviduct of laying hens, resulting in an improvement in egg albumen quality. The yolk color score was increased by both PSD and FPSD supplementation, which may be related to flavonoids, such as luteolin and apigenin (20), which may affect pigment deposition in egg yolk. However, it should be noted that there was a decreasing trend in the laying rate and albumen quality when FPSD was increased to 9%, which indicates that excessive FPSD may lead to decreased performance.

With the appeals for antibiotic-free production and attention to natural feed additives, various natural plants have been used as dietary supplements to enhance the immune performance of poultry (27, 28). In the present study, PSD had no positive effects on immune function, and birds fed 5% PSD had the lowest antibody titers against H7N9 and ND during the most experimental period, indicating a relatively weak effect on immune performance. The discrepancy might be partly due to its constituents and antinutritional factors, such as trypsin inhibitor, phytic acid, and erucic acid. Notably, the antibody levels of H7N9 and ND were significantly higher in the FPSD groups at some stages than in the control, indicating the improved immunity of FPSD-treated birds, which was probably because the PSD had been fermented. The microbial fermentation process can produce many beneficial substances, such as small-size peptides, exoenzymes, vitamins, and organic acids, which can promote the immunity of animals (29). Similar results were reported by Wang et al. (30), who found that serum IgM and IgG of birds fed a fermented cottonseed meal diet were greater than those fed an untreated cottonseed meal diet. The relative weights of the immune organs (e.g., spleen and thymus gland) and serum Glb of the broilers fed with 5% and 10% fermented corn gluten meal were higher than those fed with untreated corn gluten meal (31), suggesting that fermented feeds could promote immune organ growth and development.

Blood serum biochemistry parameters reflect the physiological and metabolic status of birds and are influenced by numerous factors, among which diet composition is one of the most important. T-CH, TGs, LDL, and HDL are related to lipid metabolism, which is important for health status. GSH-Px and SOD are usually considered antioxidant indices that reflect the antioxidant status of animals, while MDA is the main final product of lipid peroxidation and has often been used to assess oxidative damage (32). Previous studies reported that dietary supplementation with natural plants or their byproducts could promote lipid metabolism in birds. For example, increasing the level of dietary sunflower seed meal increased the serum HDL concentration and decreased serum LDL and TG concentrations in broiler chicks (33). Dietary rubber seed oil supplementation decreased the total triglyceride and cholesterol levels of egg yolks in laying hens (5). In the present study, birds in the PSD and FPSD groups had lower serum TGs and LDL and higher serum HDL than those in the control groups, especially for the greater improvement associated with FPSD, which indicated superior lipid-lowering effects of FPSD. Birds fed PSD and FPSD had lower serum concentrations of MDA and higher activities of GSH-Px and SOD than those fed the control diet, which showed higher antioxidant capacity and a healthier physiological state. The enhanced antioxidant status induced by PSD and FPSD supplementation is likely due to the antioxidant compounds, as mentioned above.

The fatty acid composition plays an important role in determining the flavor of poultry meat (34), while feeding fermented feed could improve meat quality (35). For example, broiler chickens fed fermented rapeseed meal had a lower proportion of SFA and a higher proportion of UFA in thigh meat (25). Feeding fermented Ginkgo biloba leaves reduced SFAs and increased PUFAs in the breast muscle of broiler chickens (36). Similar results were found in the present study, in which FPSD supplementation could effectively change the fatty acid composition of meat in laying hens. The total SFA content in breast and thigh meat was reduced greatly with dietary supplementation with 5% PSD and FPSD, which was primarily caused by the decrease in C14:0 and C16:0. The decrease in SFA in the muscles of broiler chickens as a result of dietary PSD and FPSD may have an effect on lipid metabolism that led to a reduction in serum TG concentration, as mentioned above. Dietary 5% PSD and FPSD reduced the total MUFA content in the thigh and breast (except for in the 5% FPSD group), which was primarily caused by the decrease in C18:1n-9. However, the PUFA content in both the breast and thigh muscles of laying hens was improved by dietary 5% PSD and any level of FPSD, which primarily caused by increasing LA, ALA, and DHA. Among the FPSD treatment groups, the content of n-3 and n-6 PUFAs in the meat linearly increased with increasing FPSD levels, which might have been influenced by the abundant PUFAs in the FPSD, and the antioxidant properties of FPSD (polysaccharides or peptides) might contribute significantly to protecting the peroxidation of oxidative-labile PUFAs rather than that of more stable SFAs in meat (17, 18). In addition, a greater increase in ALA and DHA in the breast muscle was caused by FPSD; ALA, and DHA cannot be synthesized in the body but is required for the maintenance of optimal human health and nutrition (37), indicating the positive effects on meat flavor and quality of FPSD. However, some unsaturated fatty acids, especially AA, ALA, and DHA, are deposited differently in breast muscle and thigh muscle, which may be attributed to the different nutritional regulation in breast and thigh muscles.

Increasing interest in the fatty acid composition of yolks and their oxidative stability as it relates to human health has led to research exploring how these can be affected through dietary manipulation (7). In the present study, 5% PSD and FPSD reduced the total SFA content in egg yolk compared to the control, which was primarily caused by the decrease in C14:0 and C16:0, which showed the same lipid-lowering effects as that observed for the meat. In addition, the total MUFA content in egg yolk was decreased with increased dietary supplementation with FPSD, which was primarily caused by the decrease in C16:1 and C18:1n-9 and was similar to results found by Petrović et al. (38), that yolk C18:1n-9 content was significantly decreased with the amount of linseed oil in feed. The content of yolk C18:1n-9 decreased linearly, while the content of C18:2n-6 increased linearly with increasing FPSD levels in diets, which might be because hens were able to convert C18:1n-9 into C18:2n-6 via desaturation. Many researchers have found that the content of AA in eggs is strongly affected by high PUFA levels in feed (38, 39). However, our study showed a different result, and dietary FPSD had no effect on the AA level, which was in agreement with a previous study (5).

ALA is converted into EPA and DHA with two-step dehydrogenation by Δ-6-desaturase, followed by the addition of two carbons by an elongase, but the conversion efficiency is limited (7). This is caused by competition for the enzymes involved. The increase in amounts of n-3 PUFAs in the egg yolk was paralleled by a decrease in n-6 PUFA, especially LA and AA, which relate to competition for the desaturase enzyme needed for ALA conversion (7). Nevertheless, such competition did not decrease the amount of n-3 PUFA formation in our study; both the content of n-6 PUFAs (LA) and n-3 PUFAs (ALA, DHA) increased linearly with increasing dietary FPSD levels, while yolk EPA content remained at a low level. This might be because the increasing LA was not efficiently converted to AA in the egg yolks of our experimental birds, while the increasing ALA led to more conversion to DHA than EPA. It can be inferred that dietary FPSD could lead to a more effective conversion of ALA into DHA through competing for the enzymes with LA and AA. There is currently a strong focus on reducing the ratio of n-6/n-3 PUFA in the human diets that could reduce the risk of chronic diseases, such as obesity, cardiovascular diseases, and certain forms of cancer, as well as improve brain development and function (39, 40). In our study, the yolk n-6/n-3 ratio decreased with increasing FPSD levels in diets, and the main n-3 PUFA was DHA. Thus, it is feasible to produce n-3 PUFA (especially DHA)-enriched eggs, which are safe for adults and infants to consume *via* dietary FPSD supplementation for laying hens. Furthermore, the lowest ratio (7:12) was obtained in the 5% PSD group, since the contents of n-3 and n-6 PUFAs were significantly lower than those in the FPSD groups, which also showed positive health effects.

## 5. Conclusion

**Figure 2:**
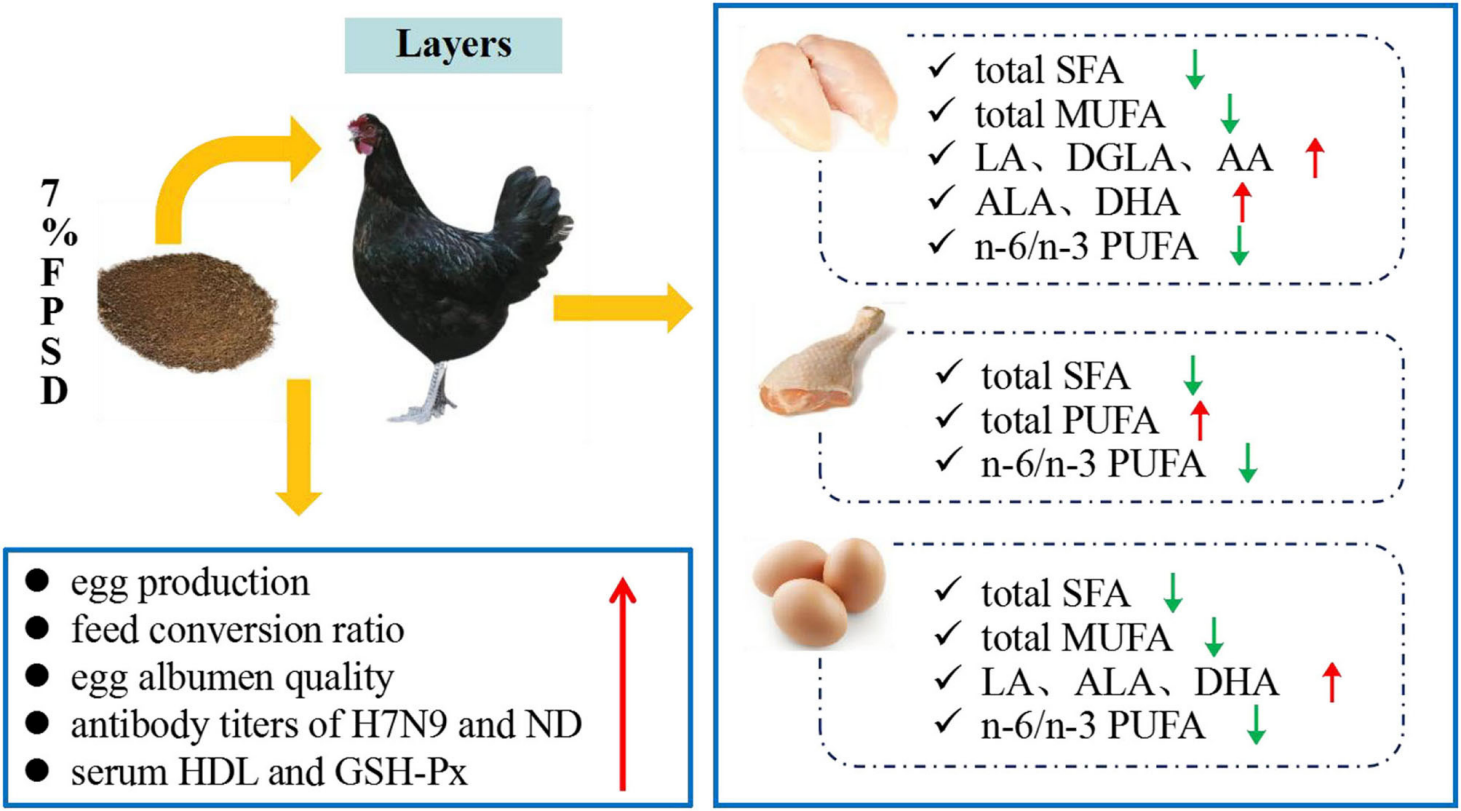
Effect of dietary 7% FPSD on the productive performance of laying hens and the fatty acids contents in meat and egg yolks. FPSD, fermented peony seed dreg.

Dietary PSD and FPSD in Xinyang laying hens enhanced the antioxidative status and enriched meat and egg yolk with n-3 PUFAs, which contributed to an improvement in the fatty acid composition, particularly with regard to the lower SFA contents and the lower n-6/n-3 PUFA ratio. However, dietary PSD had no obvious influences on hen laying performance or egg quality, while appropriate dietary FPSD was superior to PSD in improving laying rate, albumen quality, immune status, and lipid metabolism. This result suggested that up to 7% FPSD can be utilized as a feed resource in laying hen diets to produce n-3 PUFA-enriched meat and eggs and result in positive effects on production performance and health status, which could contribute to improving human health and well-being (Figure 2). Further studies are needed to explore the optimum dosage of FPSD in feed for different chicken strains.

## Data availability statement

The original contributions presented in the study are included in the article/supplementary material, further inquiries can be directed to the corresponding author.

## Ethics statement

The animal study was reviewed and approved by Committee for the Care and Use of Experimental Animals at Anhui Academy of Agricultural Science under permit no. A11-CS06. Written informed consent was obtained from the owners for the participation of their animals in this study.

## Author contributions

YW and KZ conceived and designed the study. RM, RQ, and JLu participated in the analysis of the data. ZW and QM created the model and developed the tools. WL, JLi, and YL performed the experiments and helped to revise the manuscript. YW wrote the manuscript. All authors read and approved the manuscript.
